# Development of a Real-Time Pectic Oligosaccharide-Detecting Biosensor Using the Rapid and Flexible Computational Identification of Non-Disruptive Conjugation Sites (CINC) Biosensor Design Platform

**DOI:** 10.3390/s22030948

**Published:** 2022-01-26

**Authors:** Dustin D. Smith, Joshua P. King, D. Wade Abbott, Hans-Joachim Wieden

**Affiliations:** 1 Alberta RNA Research and Training Institute (ARRTI), University of Lethbridge, Lethbridge, AB T1K 3M4, Canada; dustin.smith@uleth.ca (D.D.S.); joshdpking@gmail.com (J.P.K.); 2 Department of Chemistry and Biochemistry, University of Lethbridge, Lethbridge, AB T1K 3M4, Canada; Wade.Abbott@agr.gc.ca; 3 Lethbridge Research and Development Centre, Agriculture and Agri-Food Canada, Lethbridge, AB T1J 4B1, Canada; 4 Department of Microbiology, University of Manitoba, Winnipeg, MB R3T 2N2, Canada

**Keywords:** computational biosensor design, molecular dynamics, fluorescence, rapid kinetics, carbohydrate detection, oligogalacturonides, TogB, YePL2b, YeGH28

## Abstract

Fluorescently labeled, solute-binding proteins that change their fluorescent output in response to ligand binding are frequently used as biosensors for a wide range of applications. We have previously developed a “Computational Identification of Non-disruptive Conjugation sites” (CINC) approach, an in silico pipeline utilizing molecular dynamics simulations for the rapid design and construction of novel protein–fluorophore conjugate-type biosensors. Here, we report an improved in silico scoring algorithm for use in CINC and its use in the construction of an oligogalacturonide-detecting biosensor set. Using both 4,5-unsaturated and saturated oligogalacturonides, we demonstrate that signal transmission from the ligand-binding pocket of the starting protein scaffold to the CINC-selected reporter positions is effective for multiple different ligands. The utility of an oligogalacturonide-detecting biosensor is shown in Carbohydrate Active Enzyme (CAZyme) activity assays, where the biosensor is used to follow product release upon polygalacturonic acid (PGA) depolymerization in real time. The oligogalacturonide-detecting biosensor set represents a novel enabling tool integral to our rapidly expanding platform for biosensor-based carbohydrate detection, and moving forward, the CINC pipeline will continue to enable the rational design of biomolecular tools to detect additional chemically distinct oligosaccharides and other solutes.

## 1. Introduction

Biosensors are analytical devices that use biological components to detect target molecules and produce a measurable output. A popular approach to biosensor design is the conjugation of a fluorescent group to a solute-binding protein that harbors specificity for the target molecule, exploiting ligand-induced fluorescence changes in the protein–fluorophore conjugate to detect a target [1,2]. These protein-based biosensors have been utilized for amino acid detection [3,4,5,6], anion or cation detection [4,7,8,9,10], nucleotide detection [11,12,13], and carbohydrate detection [4,14,15,16,17,18,19]. Protein-based biosensors are often constructed by placing a fluorescent group in close proximity to the ligand-binding site, exploiting ligand-binding events to change the local environment probed by the fluorescent group. However, the placement of the fluorescent group in the vicinity of the ligand-binding site can interfere with ligand binding, thereby reducing its binding affinity or altering ligand specificity (e.g., as in [2,4,20,21]). Further innovation is, therefore, required to move beyond these trial-and-error-based approaches, which are laborious and often disrupt the underlying binding properties of the protein scaffold.

To this end, we have previously developed CINC, an in silico screening step for the rational selection of fluorophore conjugation sites distal from the ligand-binding site [18]. CINC was originally designed based on observations in our previous work that the binding of ligands to their respective binding sites altered the localized dynamics at distal sites [22,23,24,25]. CINC utilizes the explicit solvent molecular dynamics simulations of proteins in their *apo* and ligand-bound state to identify changes in localized dynamics as a consequence of ligand binding, and maps these changes to the corresponding amino acids. Changes in amino acid dynamics in response to an input (i.e., ligand binding) are calculated and ranked using our in-house developed “*F_Score_*” algorithm [18]. The aforementioned changes in amino acid dynamics upon ligand binding are then exploited for biosensor design via the placement of a fluorescent group at the selected site. To generate candidate biosensors, selected amino acid sites are substituted with cysteine and subsequently conjugated with a thiol-reactive fluorophore. CINC was previously used to develop protein-based biosensors for the detection of maltooligosaccharides [18], and an evaluation of the efficacy of prior design considerations has since been used to improve our scoring algorithm. Here, we present a streamlined and improved version of the CINC pipeline scoring algorithm (*F_Score2.0_*), informed by computational and experimental data in our previous report [18]. Our prior study with CINC examined ligand-induced changes in Root Mean Square Fluctuation, changes in distance to tryptophan, changes in solvent accessibility, changes in proximity to ligand, and changes in backbone dihedral angles when scoring candidate fluorophore conjugation sites [18]. However, experimental data suggested that the examination of changes in backbone dihedral angle dynamics were critical to successful biosensor design and could alone be used to improve the efficacy of CINC designs [18], but this hypothesis had not been tested. The *F_Score2.0_* ranking algorithm emphasizes changes in the backbone dihedral angle dynamics when scoring potential candidate biosensors to improve the efficacy of our design pipeline. We then use CINC to engineer TogB from *Yersinia enterocolitica* [26] for the direct, specific, and continuous monitoring of both saturated and 4,5-unsaturated oligogalacturonides. In addition to testing the efficacy of CINC, the use of TogB as a protein scaffold in the CINC pipeline presents a unique biosensor engineering challenge with respect to the ligand-binding properties of TogB. Specifically, we investigate if CINC-detected changes in amino acid dynamics are consistent when the protein scaffold binds chemically distinct ligands within the same binding pocket. Such information will be useful for applications where the desired biosensors not only maintain the ligand-binding properties of the protein scaffold, but also must be capable of faithfully reporting the binding of the whole ligand range via signal transmission from the ligand-binding site to the reporter, producing a detectable output.

## 2. Methods

*Molecular dynamics simulations.* Structural models of TogB in its *apo*, digalacturonic acid (digalUA)-bound state, 4,5-unsaturated digalacturonic acid (unsatdigalUA)-bound state, and trigalacturonic acid (trigalUA)-bound states were obtained from a protein databank (2UVG, 2UVH, 2UVI, and 2UVJ, respectively [26]). Each protein structure was solvated using a TIP3P water box, extending 10 Å from all atoms in the protein, and the system was neutralized with Na^+^ ions. The potential energy of water was minimized using AMBERFF99S for 10,000 steps, followed by a potential energy minimization of the entire system using AMBERFF99S and GLYCAM force fields [27,28,29]. Subsequently, the system was heated to 300 K for 10,000 steps, and molecular dynamics simulations were performed using the AMBER suite [29] for 100 ns (3 replicates each for *apo*, unsatdigalUA-bound, digalUA-bound, and trigalUA-bound states) at 2 fs step size, where temperature was maintained using Langevin dynamics. Trajectories were combined using cpptraj [30], and the Root Mean Squared Deviation (RMSD) for both *apo* and ligand-bound states of TogB remained stable after an initial equilibration (Figure S1).

*Adaptation of CINC and development of F_Score2.0_.* The CINC pipeline examines small-scale changes in the dynamic features of amino acid positions that impact a conjugated fluorophore. By design, the *F_Score_* ranking system is flexible and can weigh changes in the aforementioned criteria differently, guided by wet-lab data. Because CINC is based on first principles, it has broad utility for applications (e.g., custom biosensor design) where high-resolution structural data of an input protein scaffold are available. Results of our previous study suggested that changes in the dynamics of backbone dihedral angles between the *apo* and ligand-bound state may alone be used as a metric for producing effective biosensors [18]. As well, our prior study using CINC had not examined algorithm predictive ability and consistency in cases where the protein scaffold bound multiple different types of ligands. To this end, the scoring algorithm used for the current study focuses entirely on changes in backbone dihedral angle dynamics, and data were examined for the *apo* vs. various different ligand-bound states of TogB. Backbone dihedral angles were calculated using VMD [31] by determining ϕ (between atoms C-N-Cα-C) and ψ (between atoms N-Cα-C-N) angles throughout each simulation, constructing a Ramachandran plot for each amino acid position. Each Ramachandran plot was transformed into a 180 × 180 matrix, and the sum of the absolute differences between the ligand-bound (*B_Lr_*) and *apo* (*B_Ar_*) matrices were determined for each amino acid position, resulting in a single value for each position representing the difference between the *apo* and ligand-bound state plot (performed using in-house developed R scripts). Values were normalized by dividing by the largest difference value in the data set, resulting in *F_Score2.0_* values ranging from 0 to 1 (Equation (1)). For TogB, amino acids directly involved in binding site interactions with ligand [26] were not considered to be candidates for fluorophore conjugation. *F_Score2.0_* values from each replicate were averaged, and averages, as well as individual replicates, were plotted using GraphPad Prism v. 9.0 (GraphPad Software).

(1) $$F_{score2.0}=\frac{\sum \left|\left[B_{Lr}\right]-\left[B_{Ar}\right]\right|}{v\sum \left|\left[B_{Lr}\right]-\left[B_{Ar}\right]\right|}$$

*Construct design.* All *togB* genes were engineered to lack the signal secretion peptide found in wild-type *togB*, as previously described [26]. Variants of *togB* were synthesized with flanking 5′ NdeI and 3′ XhoI restriction sites, then subcloned into pET28a, yielding pET28a::*togB K99C*, pET28a::*togB F247C*, pET28a::*togB A284C*, pET28a::*togB K362C*, and pET28a::*togB D363C* (BioBasic Canada Inc., Markham, ON, Canada). Genes for *yePL2b* and *yeGH28* were subcloned into the 5′ NheI and 3′ XhoI sites of pET28a, yielding pET28a::*yePL2b* and pET28a::*yeGH28*.

*Overexpression and purification of TogB variants.* LB + kanamycin media (10 g/L tryptone, 5 g/L yeast extract, 10 g/L NaCl, 50 μg/mL kanamycin) was inoculated to an optical density of 600 nm (OD_600_) ≈ 0.1 with *Escherichia coli* BL21(DE3) gold cells transformed with pET28a::*togb* variants. Cultures were incubated at 37 °C with 200 RPM shaking until OD_600_ ≈ 0.6, then cooled to 16 °C at 200 RPM shaking for one hour prior to induction with 300 µM isopropyl-β-D-thiogalactopyranoside (IPTG). Cultures were grown at 16 °C with 200 RPM shaking for 16 h prior to harvesting via centrifugation (5000× *g*, 15 min, 4 °C).

Cells were resuspended in 7 mL of Buffer A per gram of cells (Buffer A: 20 mM Tris-Cl (pH 8.0 at 4 °C), 500 mM NaCl, 10 mM imidazole, 10% glycerol, 7 mM β-mercaptoethanol, 1 mM phenylmethylsulfonylfluoride). Lysozyme was added to a final concentration of 1 mg/mL, and the cell suspension was incubated on ice for 30 min with periodic inversion. The cell suspension was then mixed on ice for 5 min with 12.5 mg sodium deoxycholate per gram of cells. The mixture was sonicated (Branson Sonifier 450, Danbury, CT, USA) for 30 s at 50% output and 50% duty cycle (repeated once, with a 5 min break between cycles). Insoluble cellular debris was pelleted via centrifugation (3000× *g*, 30 min, 4 °C), followed by further centrifugation of the supernatant to collect the S30 fraction (30,000× *g*, 45 min, 4 °C). S30 supernatant was loaded onto a 5 mL gravity flow column with Ni^2+^ Sepharose IMAC resin (GE Lifesciences) equilibrated with Buffer A. The column was subsequently washed three times with 3 column volumes of Buffer A, and four times with 3 column volumes of Buffer B (Buffer A with 20 mM imidazole) to remove weakly bound proteins. TogB protein variants were eluted in 5 mL fractions using Buffer C (Buffer A with 250 mM imidazole) and analyzed by Sodium Dodecyl Sulfate Polyacrylamide Gel Electrophoresis (SDS-PAGE) with Coomassie Brilliant Blue staining. Fractions containing TogB variants were pooled, and buffer exchanged to Buffer D (20 mM Tris-Cl pH 8.0 at 4 °C, 30 mM imidazole, 500 mM NaCl, 10% glycerol) using VivaSpin 20 concentrator columns with a 10 kDa molecular weight cut-off (GE Lifesciences, 15 mL Buffer D added to 5 mL sample, concentrated to 5 mL, repeated 3 times). Purification yields were typically 20–100 mg of protein per liter of culture, and purity was typically >95%, based on ImageJ [32] densitometry analysis of Coomassie Brilliant Blue-stained SDS-PAGE.

*Overexpression and purification of YePL2b and YeGH28.* The overexpression of YePL2b and YeGH28 was achieved using the same procedure as the overexpression of TogB variants, except the final concentration of IPTG used for induction was 200 µM [33]. The purification procedure for YePL2b and YeGH28 was the same as the purification of TogB variants, except fractions in Buffer C were buffer exchanged into Buffer E (20 mM Tris-Cl (pH 8.0 @ 20 °C)) using dialysis (30 mL sample in 500 mL Buffer E, 4 changes, molecular weight cut-off 12.4 kDa Sigma PN: D0530). Purification yields were typically 60–100 mg per liter of culture, and purity was typically >95%, based on ImageJ [32] densitometry analysis of Coomassie Brilliant Blue-stained SDS-PAGE. Protein concentrations were determined using extinction coefficients *ε_280, YePL2b_* = 114,835 M^−1^·cm^−1^, and *ε_280, YeGH28_* = 68,425 M^−1^·cm^−1^, calculated from primary sequence data using ExPASy ProtParam [34]. Purified proteins were flash frozen in liquid nitrogen and stored at −80 °C for future use.

*Fluorescent labeling of TogB variants.* TogB variants (100 µM concentration in labeling reaction, 5 mL labeling reaction volume) were each incubated with 2 mL Ni^2+^ Sepharose IMAC resin in Buffer D. Then, 7-Diethylamino-3-[N-(2-maleimidoethyl)carbamoyl]coumarin (MDCC; Sigma PN: 05019; 25 mM stock in dimethylformamide) was added at five-fold molar excess to each TogB variant, corresponding to a 500 µM concentration in the labeling reaction. Labeling reactions were subsequently incubated at 4 °C for 16 h in an end-over-end mixer. Mixtures were centrifuged (500× *g*, 2 min, 4 °C) to collect Ni^2+^ Sepharose IMAC resin, and the supernatant was removed. Ni^2+^ Sepharose IMAC resin was washed six times with three resin volumes of Buffer D (500× *g*, 2 min per wash, 4 °C). Bound protein was eluted six times (500× *g*, 2 min, 4 °C) using 1 resin volume of Buffer F (Buffer D with 250 mM imidazole) per fraction. Samples from the labeling procedure were examined using SDS-PAGE, and the resulting gels were imaged (460 nm light, Cy2 Filter, Amersham Imager 600, GE Lifesciences) to confirm the presence of the MDCC label prior to staining with Coomassie Brilliant Blue. Fractions containing the desired protein–fluorophore conjugate were pooled and dialyzed into Buffer G at 4 °C (50 mM Tris-Cl (pH 8.0 @ 20 °C), 500 mM NaCl, 10% glycerol; 4 mL sample into 500 mL Buffer G, 3 changes, molecular weight cut-off 12.4 kDa Sigma PN: D0530). The recovery of protein from the labeling procedure was commonly ~50%, and labeling efficiency ranged from 60 to 80%. Concentrations of MDCC-conjugated TogB variants were determined using spectrophotometry and using Equations (2)–(4). Parameters used were as follows: *A*_280_ is the absorbance at 280 nm, *A*_430_ is the absorbance at 430 nm*, ε_280, TogB_* = 90,300 M^−1^·cm^−1^ is the extinction coefficient of TogB at 280 nm, calculated using ExPASy ProtParam and based on the protein primary sequence [34], 0.164 is used to correct for MDCC absorbance at 280 nm [20], *L* is the instrument pathlength in cm, and *ε_430, MDCC_* = 46,800 M^−1^·cm^−1^ [20]. Purified protein–fluorophore conjugates were flash frozen in liquid nitrogen and stored at −80 °C for future use. Preparations of the TogB D363C-MDCC Biosensor (PN: B1003) and custom biosensor design services using CINC are currently available from Allos Bioscience Ltd.

(2) $$\left[TogB\right]=\frac{A_{280}-\left(A_{430}\times 0.164\right)}{\varepsilon_{280,\;TogB}\times L}$$

(3) $$\left[MDCC\right]=\frac{A_{430}}{\varepsilon_{430,MDCC}\times L}$$

(4) $$Labeling\;Efficiency=\frac{\left[MDCC\right]}{\left[TogB\right]}\times 100\%$$

*Carbohydrates.* UnsatdigalUA was produced using methods similar to those described previously [26,35]. Polygalacturonic acid (PGA, PN:P-PGACT, Megazyme) was dissolved in water at 20 mg/mL and dialyzed into water to remove small carbohydrate impurities (3.5 kDa molecular weight cut-off (Spectra/Por3), 50 mL sample into 2 L water, 2 changes). A 50 mL solution of 10 mg/mL PGA was digested overnight at 20 °C with 1 μM YePL2b exopolygalacturonate lyase in 1 mM Tris-Cl (pH 8.0). The sample solution was evaporated to dryness using a SpeedVac, and re-dissolved in 2 mL water followed by the addition of 8 mL of ethanol and 0.5 mL of acetic acid. The tube was then stored at 4 °C for 24 h, followed by centrifugation (14,000× *g*, 10 min, 20 °C). The pellets were washed with the same acidified ethanol solution and centrifuged (14,000× *g*, 10 min, 20 °C). Supernatants from the two centrifugations were pooled and evaporated to dryness using SpeedVac. Reaction products were examined via Thin Layer Chromatography using Silica 60 plates (Millipore) to confirm the production of unsatdigalUA (mobile phase 2:1:1 butanol: acetic acid: water, plates stained in 1% orcinol (PN: O1875, Sigma) in 70:3 ethanol: sulfuric acid, and removed from stain and heated using Bunsen burner). Concentrations of unsatdigalUA were determined by mass (FW = 352.3 g/mol [26]), and confirmed in solution using spectrophotometry and *ε_230, unsatdigalUA_* = 5 200 M^−1^·cm^−1^ [35,36]. Preparations of unsatdigalUA are currently available from Allos Bioscience Ltd. (PN: C1002). TrigalUA (PN: T7407) and galacturonic acid (PN: 48280) were purchased from Sigma, and digalUA (PN: O-GALA2) was purchased from Megazyme.

*Equilibrium fluorescence measurements.* A Quanta Master 60 Fluorescence Spectrometer was utilized for fluorescence spectrophotometry measurements (Photon Technology International; excitation wavelength 420 nm, emission wavelength 440–520 nm, excitation slit widths: 3 nm, emission slit widths: 6 nm, step size: 1 nm, integration: 1 s). All equilibrium binding measurements were performed in Buffer H (50 mM Tris-Cl (pH 8.0 @ 20 °C), 500 mM NaCl) at 20 °C with MDCC-conjugated TogB variants at a concentration of 100 nM. Carbohydrate concentrations were at least three-fold higher than the previously reported affinity (*K_D_*) values describing binding to TogB [26], and were as follows: unsatdigalUA: 16 μM, digalUA: 48 μM, trigalUA: 570 μM. Fluorescence emission spectra were plotted using GraphPad Prism v. 9.0 (GraphPad Software).

*Rapid kinetics measurements.* A KinTek SF-2004 (Kintek Corp., Snow Shoe, PA, USA) rapid mixing device (stopped-flow apparatus) was used to collect rapid kinetics data. Experiments were performed at 20 °C, fluorescence was excited at 420 nm, and fluorescence emissions were detected after a 450 nm long-pass filter (NewPort Corp., Irvine, CA, USA). All experiments in the stopped-flow apparatus were performed in Buffer H. Individual fluorescence time courses were fit with a one-exponential function (Equation (5)), or a two-exponential function (Equation (6)), where *F* is the fluorescence observed at time *t*, *F_∞_* is the final fluorescence, *A* the signal amplitude, and *k_app_* the apparent rate (TableCurve, Systat Software). To obtain *K_D_* values, a hyperbolic function was fit to the data using GraphPad Prism v. 9.0 (Equation (7)), where *Y* indicates ligand binding at a given *[carbohydrate]* and *B_max_* indicates maximum plateau value.

(5) $$F=F_{\infty }+A_{1}\times \exp\left(-k_{app1}t\right)$$

(6) $$F=F_{\infty }+A_{1}\times \exp\left(-k_{app1}t\right)+A_{2}\times \exp\left(-k_{app2}t\right)$$

(7) $$Y=\frac{B_{max}\times \left[carbohydrate\right]}{K_{D}+\left[carbohydrate\right]}$$

## 3. Results

**Figure 1:**
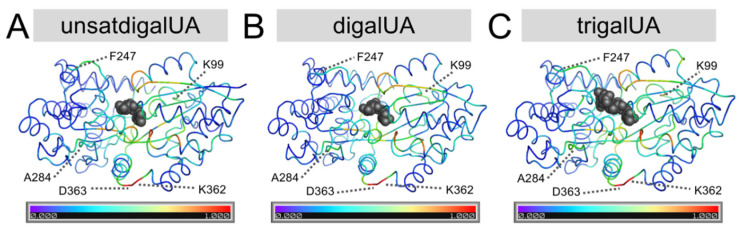
Small-scale changes in amino acid dynamics upon ligand binding identified using *F_Score2.0_*. Average *F_Score2.0_* values for each *apo* vs. ligand-bound state are projected onto their corresponding PDB structures: TogB bound to unsatdigalUA ((**A**), PDB 2UVI), TogB bound to digalUA ((**B**), PDB 2UVH), and TogB bound to trigalUA ((**C**), PDB 2UVJ). Ligand is shown using grey spheres, and protein backbone is shown as a ribbon, coloured according to *F_Score2.0_* values. Candidate labeling positions selected based on *F_Score2.0_* values are also indicated.

**Figure 2:**
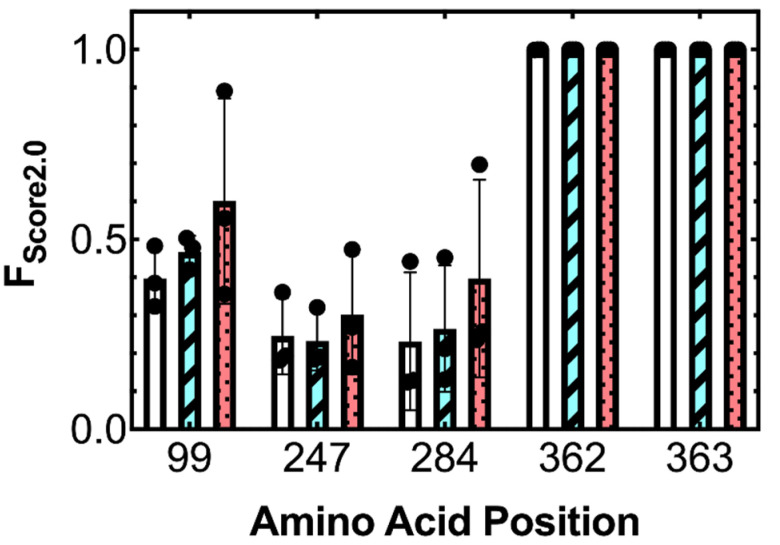
*F_Score2.0_* values reflecting changes in dynamics of backbone dihedral angles at candidate labeling positions. Changes in dynamics of candidate labeling positions relative to dynamics in the TogB *apo* state are shown for TogB∙unsatdigalUA (white bars), TogB∙digalUA (cyan striped bars), and TogB∙trigalUA (red dotted bars). Each bar reflects the results of 3 replicates ± s.d. with results from individual molecular dynamics simulations superimposed on the plot (black dots).

*Use of CINC to select fluorophore conjugation positions in TogB.* TogB *apo*, TogB-unsatdigalUA, TogB-digalUA, and TogB-trigalUA were each subjected to 100 ns molecular dynamics simulations in triplicate and analyzed using the CINC pipeline to determine amino acid *F_Score2.0_* values. Small-scale changes in amino acid dynamics in the *apo* vs. ligand-bound states of TogB were evident when using *F_Score2.0_*, with several mid- to high-scoring positions distal from the ligand-binding site (Figure 1). The conjugation of a fluorescent group distal to the ligand-binding site is preferred to the modification of the binding site, as fluorophore conjugation near the binding pocket can lead to proteins with altered or reduced ligand affinity and/or specificity (e.g., as in [4]). To validate the robustness of *F_Score2.0_* (i.e., for use in cases that may have subtle or drastic changes in amino acid dynamics), the calculated *F_Score2.0_* values were ranked (Figure 1, data not shown), and five mid- and high-scoring positions were selected to construct the candidate biosensors labeled with a thiol-reactive fluorophore (Figure 2, Figure S2 and Figure S3). This included the highest-scoring position for TogB (363, *F_Score2.0_* = 1), which is located approximately 20 Å from the bound ligand in the unsatdigalUA-bound TogB structure (PDB 2UVI).

*Oligogalacturonide detection by candidate biosensors.* Each TogB variant was conjugated to MDCC, a diethylaminocoumarin that is commonly used in the construction of solute-binding protein-based biosensors due to its size, cost effectiveness, and low probability to affect the solubility of the conjugate [8,18,20,37,38]. The MDCC-conjugated TogB variants were then examined for their ability to detect unsatdigalUA, digalUA, and trigalUA using fluorescence spectrophotometry. Four of the five TogB-MDCC conjugates were able to selectively detect the binding of the target carbohydrates and did not alter their fluorescence in the presence of the non-specific ligand, galacturonic acid (Table 1, Figure S4 and Figure S5). To better understand the behaviour of the Biosensor as well as its scaffold protein, and dependent on the downstream application, its performance under a wide range of physicochemical conditions (e.g., altered pH, ionic strength, and temperature) or in the presence of additional analytes in complex mixtures needs to be characterized. Together, these results demonstrate that examining changes in dihedral angle amino acid dynamics in the CINC pipeline alone is effective at rapidly informing biosensor rational design and streamlining biosensor development.

**Figure 3:**
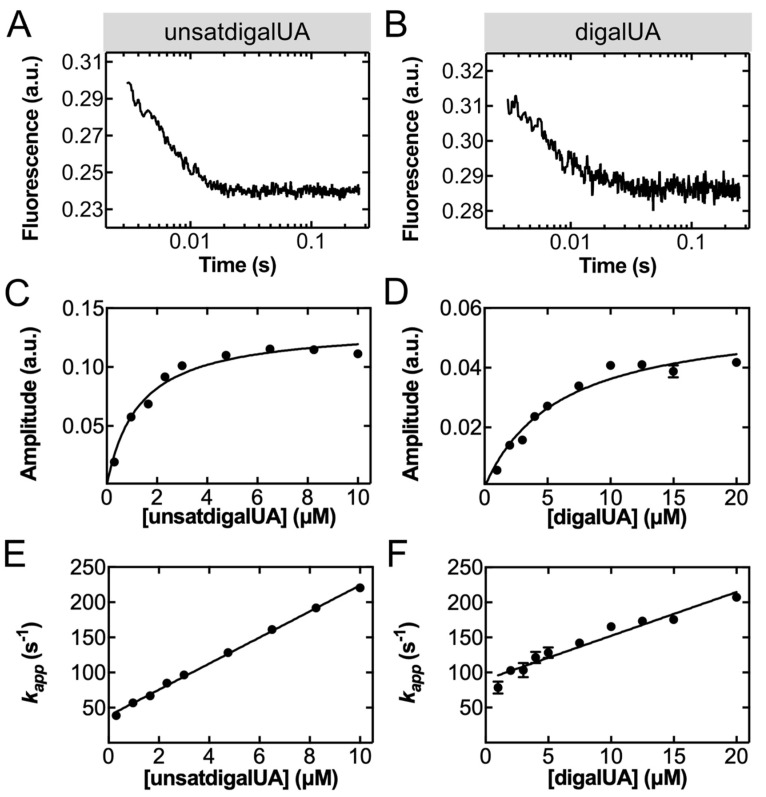
Concentration dependence of unsatdigalUA association rate and digalUA association rate. Representative fluorescence time course of 100 nM TogB D363C-MDCC binding 10 μM unsatdigalUA (**A**) or 10 μM digalUA (**B**). Fluorescence time courses were obtained for 100 nM TogB D363C-MDCC binding to ligands across a range of carbohydrate concentrations (0.3–10 μM for unsatdigalUA, and 1–20 μM for digalUA). Fluorescent time courses were fit with a one-exponential function (Equation (5)) to determine amplitude and *k_app_*. Amplitudes of signal change were plotted against concentrations of unsatdigalUA (**C**) and digalUA (**D**) and fit with a hyperbolic function (Equation (7)) to determine dissociation constant (*K_D_* = 1.3 ± 0.5 μM for unsatdigalUA, *K_D_* = 6 ± 1 μM for digalUA). *k_app_* was plotted against concentrations of unsatdigalUA (**E**) and digalUA (**F**) and fit with a linear function to determine association constants (*k_on_* = 18.6 ± 0.7 μM^−1^·s^−1^ for unsatdigalUA, and 6 ± 1 μM^−1^·s^−1^ for digalUA). Each data point in panels (**C**–**F**) reflects mean ± s.d. at the indicated carbohydrate concentration (n = 3).

*Rapid kinetics of unsatdigalUA and digalUA detection by TogB D363C-MDCC.* TogB D363C-MDCC, the highest CINC scorer, displayed the largest fluorescence change in response to the target carbohydrates (Table 1, Figure S4). However, for use in CAZyme characterization assays, a detailed knowledge of the underlying kinetics of ligand binding to TogB D363C-MDCC is required. Kinetic parameters for ligand binding to TogB D363C-MDCC were determined using the stopped-flow method, a rapid mixing device coupled with a fluorescence spectrophotometer which enables the real-time monitoring of biomolecular events. In agreement with equilibrium-state fluorescence data (vide supra), the mixing of TogB D363C-MDCC with unsatdigalUA (Figure 3A) or digalUA (Figure 3B) resulted in a fluorescence decrease. The resulting fluorescence time courses were best fit with a one-exponential function to obtain *A*_1_ and *k_app_* (Equation (5)). *A*_1_ values of the fluorescence time courses obtained at increasing concentrations of unsatdigalUA (Figure 3C) or digalUA (Figure 3D) were plotted against ligand concentrations, and a hyperbolic function (Equation (7)) was fit to the data to determine *K_D_* = 1.3 ± 0.5 μM for unsatdigalUA and *K_D_* = 6 ± 1 μM for digalUA. The *K_D_* value obtained for unsatdigalUA binding to TogB D363C-MDCC is similar to the affinity values for the unmodified protein determined previously via isothermal titration calorimetry (*K_D_* = 5 ± 1 μM) or UV difference spectroscopy (*K_D_* = 3.2 ± 0.1 μM) [26]. As well, the *K_D_* determined for digalUA binding to TogB D363C-MDCC is comparable to previously reported binding affinity data determined for the unmodified protein using isothermal titration calorimetry (*K_D_* = 16 ± 2 μM) and UV difference spectroscopy (*K_D_* = 11.8 ± 0.5 μM) [26]. The apparent rate values obtained from the fluorescence time courses were plotted against ligand concentrations and fit with a linear function to determine *k_on_* = 18.6 ± 0.7 μM^−1^·s^−1^ for unsatdigalUA (Figure 3E) and *k_on_* = 6 ± 1 μM^−^1·s^−1^ for digalUA (Figure 3F). TrigalUA binding to TogB D363C-MDCC was also examined using the stopped-flow method, but under the conditions, tested binding was likely too fast and occurred in the dead time of the stopped-flow apparatus (data not shown). Together, these results demonstrate that the CINC pipeline is robust in selecting fluorophore conjugation sites that do not disrupt ligand binding, and the resulting TogB D363C-MDCC biosensor is capable of rapid, sensitive ligand detection.

**Figure 4:**
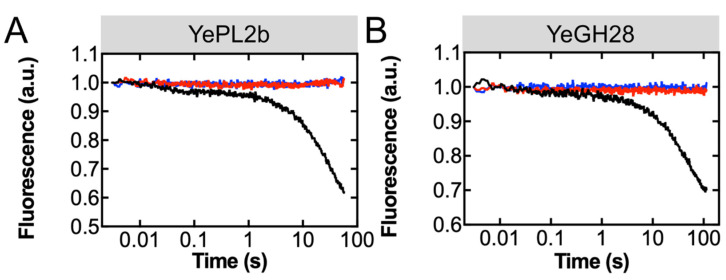
Oligogalacturonide release from CAZyme-catalyzed degradation of polygalacturonic acid detected by TogB D363C-MDCC. Representative fluorescence time courses for product released by 250 nM YePL2b ((**A**), black fluorescence time course) or 250 nM YeGH28 ((**B**), black fluorescence time course) in the presence of 0.5 mg/L PGA and detected by 250 nM TogB D363C-MDCC. Negative controls in the absence of CAZyme (red fluorescence time courses) and in the absence of PGA (blue fluorescence time courses) are shown.

*Detection of oligogalacturonide release from a polysaccharide lyase and a glycoside hydrolase.* To demonstrate the utility of the TogB D363C-MDCC biosensor for characterizing enzyme activity, we examined oligogalacturonide release from the CAZyme-catalyzed degradation of PGA in real time. YePL2b is an exo-acting polysaccharide lyase from *Y. enterocolitica* that cleaves PGA using β–elimination, producing unsatdigalUA as the major product [33,35]. Using the stopped-flow method, a solution containing TogB D363C-MDCC and YePL2b was rapidly mixed with PGA, and the resulting fluorescent time courses were recorded and best fit with a two-exponential function (Figure 4, Equation (6)). The observed fluorescence decrease represents the real-time detection of the unsatdigalUA, because in the absence of either PGA or YePL2b, the fluorescence of TogB D363C-MDCC remains unchanged over the time course of the measurement (Figure 4A). The values determined from the aforementioned data reveal a rapid initial burst phase (*k_app_*_1_ = 39 ± 8 s^−1^), consistent with the first round of product release from the CAZyme, whereas the slower second phase (*k_app_*_2_ = 0.033 ± 0.005 s^−1^) likely represents a multiple turnover phase. The multiple turnover phase is slower than the burst phase, as it requires the cleaved (now shorter) PGA to reoccupy the +1 and +2 subsites of YePL2b [35] after the cleavage of the (longer) PGA, and the dissociation of the unsatdigalUA product (Table 2).

To demonstrate that the TogB D363C-MDCC biosensor can also detect the saturated oligogalacturonide products generated by PGA hydrolysis (i.e., digalUA), we investigated the product release from YeGH28. YeGH28 is an exo-acting glycoside hydrolase from *Y. entercolitica* that hydrolyzes PGA, producing digalUA as the major product [39,40]. Using the stopped-flow method, we were able to observe, similar to YePL2b, a bi-phasic fluorescence decrease in the presence of TogB D363C-MDCC, YeGH28, and PGA that is not observed in the negative control conditions (Figure 4B). Fitting the obtained fluorescence time courses with a two-exponential function also showed an initial burst phase (*k_app_*_1_ = 33 ± 1 s^−1^), likely representing the first round of hydrolysis and product release, followed by a slower multiple turnover phase (*k_app_*_2_ = 0.021 ± 0.001 s^−1^; Table 2). Together, these findings demonstrate that TogB D363C-MDCC is a robust detection platform for both unsatdigalUA and digalUA and can be used in complex solutions to examine enzyme activity in real time.

## 4. Discussion

In this report, we expand on our carbohydrate-detecting biosensor toolkit using CINC and demonstrate the utility of their use in CAZyme activity assays. CAZymes are a group of structurally and functionally diverse enzymes that modify linkages or decorations in carbohydrates [41], and which are involved in a large number of industrial processes [42]. Improved methods for carbohydrate detection will enable a detailed kinetic analysis of CAZymes, thereby improving their implementation in industrial processes. We utilized CINC to engineer a biosensor based on TogB from *Y. enterocolitica* for the detection of the breakdown products of PGA. TogB is the solute-binding protein of an ATP-binding cassette transporter (ABC transporter), where solute binding occurs at the interface of the N- and C-terminal domains of TogB [26]. Structural studies indicate that upon solute binding, TogB undergoes a hinge–bend motion where the N- and C-terminal domains move towards each other [26]. TogB binds both unsatdigalUA and digalUA with micromolar affinity, and has weaker affinity for trigalUA [26]. The binding specificity of TogB was ideal for the construction of a biosensor to detect the breakdown products of PGA, as TogB can bind the major breakdown products produced by both polysaccharide lyases (unsaturated products, e.g., as in [33,35]) and glycoside hydrolases (saturated products, e.g., as in [39,40]). An engineered biosensor set based on TogB can therefore enable the detailed kinetic analysis of two different CAZyme classes capable of depolymerizing PGA using different catalytic mechanisms. In general, prior approaches to detect carbohydrates are limited because they are not selective (i.e., they detect many different carbohydrates in a single reaction), they do not detect in real time, requiring endpoint derivatization, and do not scale well for high-throughput techniques (e.g., require linked enzyme assays or utilize low-throughput techniques, such as mass spectroscopy) [43]. Saturated oligogalacturonide detection methods suffer from many of the aforementioned limitations [44], whereas the detection methods for unsaturated oligogalacturonides can mitigate some of these drawbacks via the absorbance-based readout of 4,5-unsaturation in the target molecule(s) [35,36]. The TogB-based biosensor circumvents all of these limitations and represents a first-in-class method for the real-time detection of the oligogalacturonides released from CAZymes.

An underlying challenge in developing biosensor-based assays for CAZyme product detection is inefficiencies in initial biosensor development. However, the CINC platform can enable the on-demand design of biosensors for specific targets based on simple protein scaffolds. The widespread availability of biosensor-based assays where the detection of specific carbohydrates occurs in real time will be transformative in elucidating and harnessing the enzymatic properties of genome-encoded CAZymes, and the CINC pipeline reported here will allow researchers to circumvent prior bottlenecks in the biosensor design process by enabling rapid computer-aided design and screening. Of the five candidate biosensors selected using CINC, four resulted in biosensors that were capable of detecting the target carbohydrates. With a success rate of 80%, CINC, using *F_Score2.0_*, outperforms the prior approaches of selecting fluorescent labeling positions based on structural data, whose designs typically respond to the desired ligand 20–30% of the time, and often result in altered binding affinity when compared to the unmodified protein (e.g., as in [4,20]). The *F_Score2.0_* algorithm also outperforms the prior version of *F_Score_*, which had a success rate of 50% [18]. Users of CINC could further improve the success rate by increasing the stringency and selecting only high-scoring candidate labeling positions based on changes in the dihedral angle dynamics. The two highest-scoring positions in this study (Position 362, *F_Score2.0_* = 0.9997 ± 0.0002; and Position 363 *F_Score2.0_* = 1) resulted in functional biosensors with large fluorescence changes in response to ligand binding. In the case of the lower-scoring variants tested (Positions 99, 247, 284), the *F_Score2.0_* values of individual simulations showed more variability than the highest scorers (Figure 2). This increased spread of *F_Score2.0_* values for the lower-scoring positions was due to partial shifts in the equilibrium between different microstates in the *apo* vs. ligand-bound simulations. For example, Position 284 largely exists in two different microstates for both the *apo* and ligand-bound simulations (Figure S3). Upon ligand binding, the occupancy of these states is partially shifted, altering the amino acid dynamics of this individual position in a subset of the single molecules. In contrast, the high *F_Score2.0_* value for Position 363 is due to a complete shift in the equilibrium of amino acid dynamics, stabilized by ligand binding (Figure S2). We therefore speculate that complete (or near-complete) shifts in microstate occupancy in *apo* vs. ligand-bound states are generally preferred to partial shifts. Additionally, it is important to note that it is difficult to speculate on the occupancy of specific microstates at ambient temperatures without molecular dynamics simulations. For example, in the case of positions 362 and 363 in TogB, the starting X-ray crystal structures demonstrate different dihedral angles when comparing the *apo* vs. ligand-bound states. However, these static X-ray crystal structures represent local energy minima and can be constrained by contacts within the crystal lattice. It was therefore critical to examine local structural dynamics and dihedral conformations in an environment that more accurately reflected physiological conditions (e.g., at room temperature and solvated), where the biomolecule was provided with additional energy and the opportunity to explore alternative conformations. Consistent with this, we did indeed observe, in several positions, additional populations of dihedral angles not present in the starting structures (e.g., see Figure S3). For some protein scaffolds, complete microstate shifts in the *apo* vs. ligand-bound state may not be present for any amino acid positions (for example, previous CINC-designed maltooligosaccharide-detecting biosensors were based on a partial shift in microstates [18]). It is therefore important to note that our work demonstrating the design of biosensors based on partial shifts in the microstate populations of *apo* vs. ligand-bound conformations will enable the construction of biosensors for a broad range of protein scaffolds.

One of the candidate TogB-based biosensors, TogB K99C-MDCC, did not detect any of the target carbohydrates in vitro (Table 1). We hypothesise that although Position 99 was a mid-range scorer, the change in backbone dihedral angles may not have positioned a conjugated fluorophore into an altered environment for enough time to produce a detectable fluorescence change (Figure 2 and Figure S6). It is important to note that the current version of CINC does not quantify residence-times in the respective sub-states (including ones with different dihedral angles), but rather reports a probability that the dihedral angles differ upon ligand binding. Because the experimentally determined fluorescence changes are ensemble measurements, this might result in the averaging of the fluorescence of the different states, preventing their detection in vitro. Of the four engineered TogB-based biosensors that reported a fluorescence change upon ligand binding, all four detected unsatdigalUA, digalUA, and trigalUA (Table 1). With prior structure-based approaches for biosensor design, the fluorescent group is often placed in close proximity to the ligand-binding site, relying on direct interactions between the ligand and fluorescent group in order to elicit a fluorescence change. Using CINC, no such direct fluorophore–ligand interactions exist, as the fluorophore is positioned distal from the ligand-binding site. Of particular importance to the design of a TogB-based biosensor is that TogB completely internalizes its target ligands upon binding [26], complicating conventional biosensor design approaches utilizing fluorophore–ligand interaction. CINC-designed biosensors are able to overcome this challenge, as they do not rely on fluorophore–ligand interactions in order to produce a fluorescence change. Instead, CINC-designed biosensors focus on changes in amino acid dynamics at locations distal from the ligand-binding site. Against this background, it was important to determine if signal transmission from the binding site to the reporter positions occurs similarly for each ligand-bound state of TogB. Specifically, the ligand-bound states of TogB differ in that they include a discriminatory interaction between the unsaturated ligand and S271 of TogB when compared to the binding of the saturated ligands, as well as an additional hydrogen bond between disaccharides and Y276 of TogB when compared to the trigalUA-bound complex [26]. Indeed, the changes in amino acid dynamics identified via CINC in the TogB *apo* vs. ligand-bound states were capable of reporting ligand binding, despite the different interactions present in the TogB∙unsatdigalUA, TogB∙digalUA, and TogB∙trigalUA complexes. Our aforementioned in silico predictions were validated with an in vitro characterization of the TogB-based biosensors and demonstrated that signal transmission from the ligand-binding site to the CINC-designed reporter sites is likely as reliable as directly sensing the bound ligand.

In addition to streamlining the in silico CINC pipeline and improving its success rate via the implementation of *F_Score2.0_*, this work further demonstrates that biosensors produced by this pipeline do not perturb the ligand-binding properties of the starting protein scaffold. The TogB D363C-MDCC biosensor was engineered based on localized amino acid dynamics changes distal from the ligand-binding site, and the engineered fluorescent–protein conjugate has a ligand-binding affinity comparable to the unmodified protein (vide supra). The ligand-binding properties of TogB D363C-MDCC are consistent with previously developed solute-binding protein-based biosensors for the detection of phosphate and maltooligosaccharides [8,18,20,45] (Table S1). The previously developed phosphate-detecting biosensor has facilitated detailed mechanistic investigations into the timing and function of phosphate release in various systems [8,46,47,48], whereas the maltooligosaccharide-detecting biosensor has enabled a detailed kinetic analysis of CAZymes that release maltooligoaccharides as part of their functional cycle [18]. In the current report, we examine unsatdigalUA release by a polysaccharide lyase (YePL2b) and digalUA release by a glycoside hydrolase (YeGH28) during PGA digestion using TogB D363C-MDCC. TogB D363C-MDCC binds unsatdigalUA and digalUA with high affinity and rapid rates. We therefore speculate that observed fluorescence decreases caused by TogB D363C-MDCC are rate limited by the availability of free oligogalacturonides in solution produced via CAZyme-catalyzed PGA degradation. Together with the previously developed maltooligosaccharide-detecting biosensors and others, the oligogalacturonide-detecting biosensors reported here are novel enabling tools integral to our emerging biosensor platform for the rapid characterization of CAZymes. Moving forward, biosensor-based assays for the detection of and discrimination between carbohydrates that may differ with minor chemical variances (e.g., degree of polymerization, chemical modification, branch points) are amenable to multiplexing and high-throughput screening assays (e.g., for the simultaneous detection of multiple carbohydrates using biosensors with different fluorescence properties). Alternatively, the design of microfluidic devices with multiple carbohydrate-detecting biosensors in succession would circumvent the need for biosensors to have distinct fluorescence properties for the detailed analysis of carbohydrate content. Such assays would have clear utility in rapid-testing experiments and enable the characterization of complex carbohydrate-containing solutions and/or metabolic processes, such as biofuel feedstock metabolism or the gut microbiome. Furthermore, this class of biosensors also provides the additional capability of distinguishing between the formation of a nascent carbohydrate and its release into bulk solution. For example, when combined with information about the chemical step of glyosidic bond cleavage (e.g., using quench flow), one can distinguish between the chemical cleavage step and the release of a product from a CAZyme site. This, in turn, would provide critical information for a detailed kinetic analysis of de-polymerization enzymes paramount to their rational design. An additional future consideration with the CINC pipeline is the incorporation of fluorescent protein(s) at one or more positions with high *F_Score2.0_* values rather than small fluorescent dyes. For example, a recent report has utilized molecular dynamics simulations to propose that the sensing ability of a FRET-based glucose-detecting biosensor is due to the careful positioning of the donor fluorescent protein [49]. It would therefore be beneficial to develop fluorescent protein-based biosensors guided by CINC for either FRET-based readouts or single-labeled biosensors using circular permuted fluorescent proteins [50].

## 5. Conclusions

The CINC pipeline is capable of detecting and exploiting small-scale changes in amino acid dynamics between the *apo* and ligand-bound states of an input protein scaffold for biosensor design. The CINC pipeline is rapid, simple, and has general applicability in the design of novel protein–fluorophore conjugates. In terms of success rates of designs, CINC using *F_Score2.0_* outperforms conventional biosensor design methods and the prior *F_Score_* algorithm used in CINC. A current limitation of CINC is its dependence on the high-resolution structural data of a protein in its *apo* and ligand-bound state. However, moving forward, the expansion of biosensor libraries can be accelerated by coupling CINC with next-generation protein modelling, such as AlphaFold [51], circumventing the current requirement for high-resolution structural data for each protein examined.

## 6. Patents

D.D.S., D.W.A., and H.J.W. are Joint Inventors on International PCT Patent Application No. CA2021/05/01/10 (Filed 30 January 2021).

## Figures and Tables

**Table 1:** Response of fluorescently labeled TogB variants to ligand. Fluorescently labeled TogB variants were incubated in the absence and presence of saturating concentrations of unsatdigalUA, digalUA, and trigalUA (saturating concentrations defined as ligand concentration at least three-fold above previously reported dissociation constants [26]). Labeled TogB variants were also incubated in the absence and presence of a non-specific carbohydrate galacturonic acid. Values reported indicate percentage change in peak fluorescence intensity after addition of ligand (n = 1).

	w/16 µM UnsatdigalUA	w/48 µM DigalUA	w/570 µM TrigalUA	w/1710 µM Galacturonic Acid
TogB K99C-MDCC	+2%	−4%	−4%	−2%
TogB F247C-MDCC	−14%	−10%	−20%	−1%
TogB A284C-MDCC	−31%	−31%	−30%	−1%
TogB K362C-MDCC	−32%	−25%	−29%	−2%
TogB D363C-MDCC	−60%	−39%	−44%	−1%

**Table 2:** Oligogalacturonide release fit parameters obtained via CAZyme-catalyzed PGA degradation. The fitting of the two-exponential function (Equation (6)) to biphasic fluorescence time courses of oligogalacturonide release shown in Figure 4 details underlying enzyme kinetic parameters. Fit parameters for a polysaccharide lyase (YePL2b) and a glycoside hydrolase (YeGH28) are reported (mean ± s.d., n = 6 replicates for each enzyme).

	*F*_∞_ (a.u.)	*A*_1_ (a.u.)	*k*_1_ (s^−1^)	*A*_2_ (a.u.)	*k*_2_ (s^−1^)
YePL2b	0.545 ± 0.02	0.05 ± 0.03	39 ± 8	0.37 ± 0.03	0.033 ± 0.005
YeGH28	0.565 ± 0.002	0.03 ± 0.01	33 ± 1	0.30 ± 0.01	0.021 ± 0.001

## Data Availability

Not applicable.

## Supplementary Materials

The following supporting information can be downloaded at: https://www.mdpi.com/article/10.3390/s22030948/s1, Supplementary Materials is available online and includes molecular dynamics data (Figures S1–S3 and S6), fluorescence spectrophotometry data (Figure S4), structural representations of carbohydrates utilized in this study (Figure S5), and comparisons of our TogB D363C-MDCC biosensor to previously reported solute-binding protein-based biosensors (Table S1).
