# Supplementary material for: Development of a Real-Time Pectic Oligosaccharide-Detecting Biosensor Using the Rapid and Flexible Computational Identification of Non-Disruptive Conjugation Sites (CINC) Biosensor Design Platform
Source: Sensors (Basel). 2022 Jan 26;22(3):948. doi: 10.3390/s22030948 (PMC8839585; doi:10.3390/s22030948)
Supplement: Supplementary file 1 [file sensors-22-00948-s001.zip › sensors-1539821-supplementary.pdf]

Supplemental Materials: Development of a real-time pectic oligosaccharide-detecting biosensor using the rapid and flexible Computational Identification of Non-disruptive Conjugation sites (CINC) biosensor design platform

Authors: Dustin D. Smith[1,2], Joshua P. King[1,2], D. Wade Abbott[2,3], and Hans-Joachim Wieden[1,2,4,#]

Affiliations:
[1]Alberta RNA Research and Training Institute (ARRTI), University of Lethbridge, Lethbridge, AB, Canada
[2]Department of Chemistry and Biochemistry, University of Lethbridge, Lethbridge, AB, Canada
[3]Lethbridge Research and Development Centre, Agriculture and Agri-Food Canada, Lethbridge, AB, Canada
[4]Department of Microbiology, University of Manitoba, Winnipeg, MB, Canada

[#] To whom correspondence should be addressed

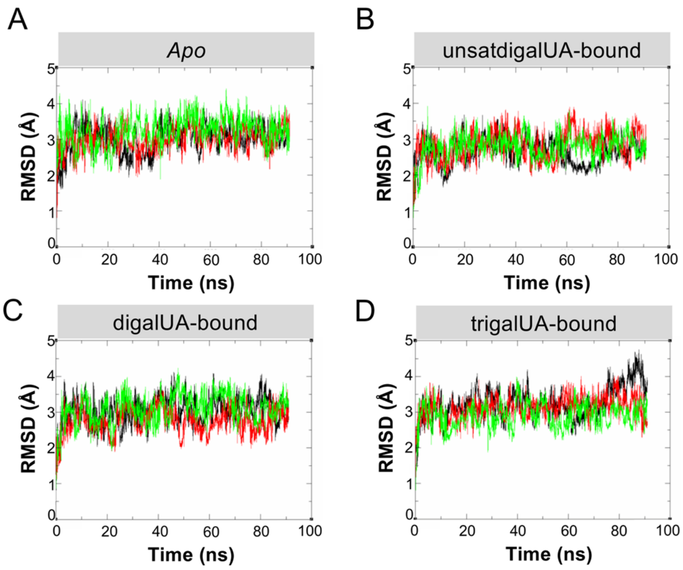

Figure S1. RMSD plots of *apo* (A), unsatdigalUA-bound (B), digalUA-bound (C), and trigalUA-bound (D) TogB for the duration of molecular dynamics simulations. Mass-weighted RMSD fits were calculated using cpptraj [30] for each successive frame in the trajectory when compared to the starting structures. The initial 10 ns of the 100 ns simulations prior to system equilibration were removed before scoring (first 10 ns not shown). Replicates for each condition (R1: Black, R2: Red, R3: Green) remain stable for

the duration of the simulations at an average RMSD of approximately 3Å from the starting structures.

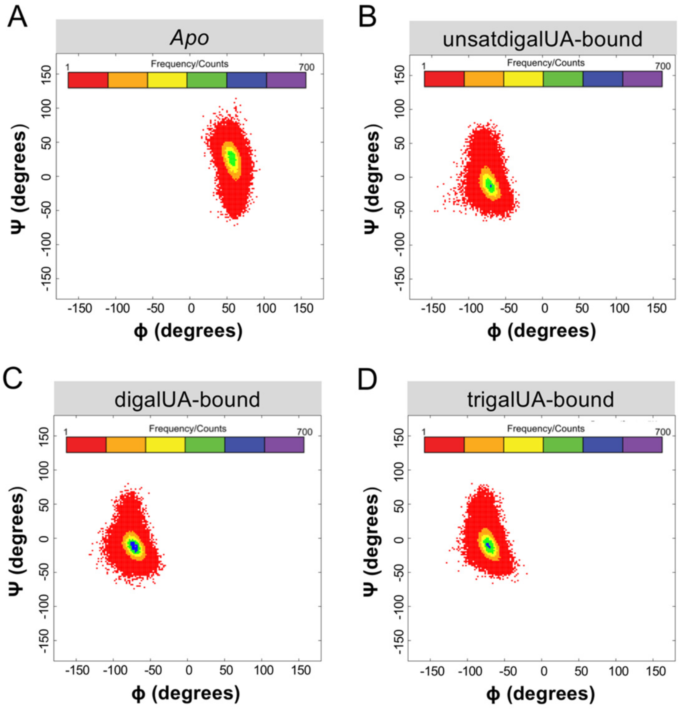

Figure S2. Amino acid position D363 ($F_{Score2.0}$ = 1) in TogB adopts unique dihedral angles in *apo* vs. ligand-bound states. Representative Ramachandran Plots of D363 during molecular dynamics simulations of TogB. Amino acid dihedral angles are shown for TogB in its *apo* state (A), unsatdigalUA-bound state (B), digalUA-bound state (C), and trigalUA-bound state (D).

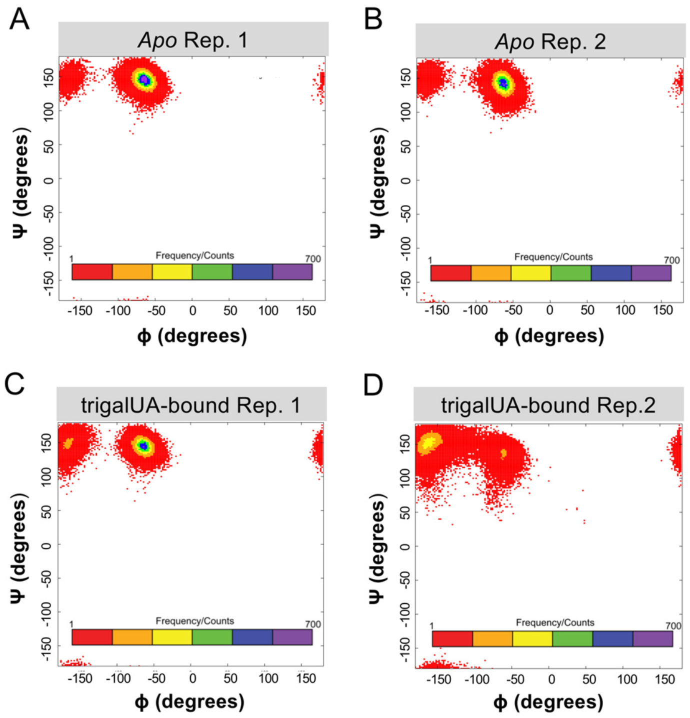

Figure S3. Amino acid position A284 ($F_{Score2.0}$ = 0.4 ± 0.3 for trigalUA) in TogB exhibits a partial shift in the equilibrium between conformations upon ligand binding. Dihedral angles in *apo* (A, B) and trigalUA-bound (C, D) states are shown for two molecular dynamics simulation replicates, and two populations of dihedral angles are present for both states. The propensity for Position 284 to exist in one population of dihedral angles vs. another is altered upon ligand binding, leading to a change in fluorescent output by the ensemble *in vitro* upon ligand binding (Table 1).

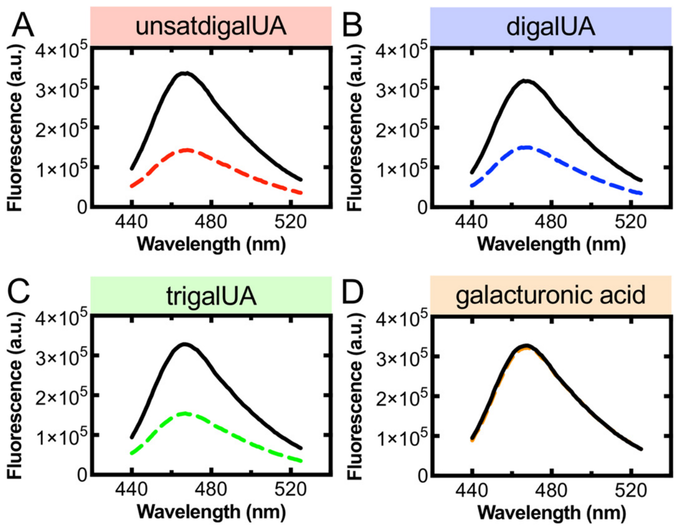

Figure S4. Representative fluorescence emission spectra of TogB D363C-MDCC response to ligand. TogB D363C-MDCC (100 nM) in the absence (black solid lines) and presence of 16 **μ**M unsatdigalUA (A, dashed red line), or 48 **μ**M digalUA (B, dashed blue line), or 570 **μ**M trigalUA (C, dashed green line), or 1710 **μ**M galacturonic acid (D, dashed orange line).

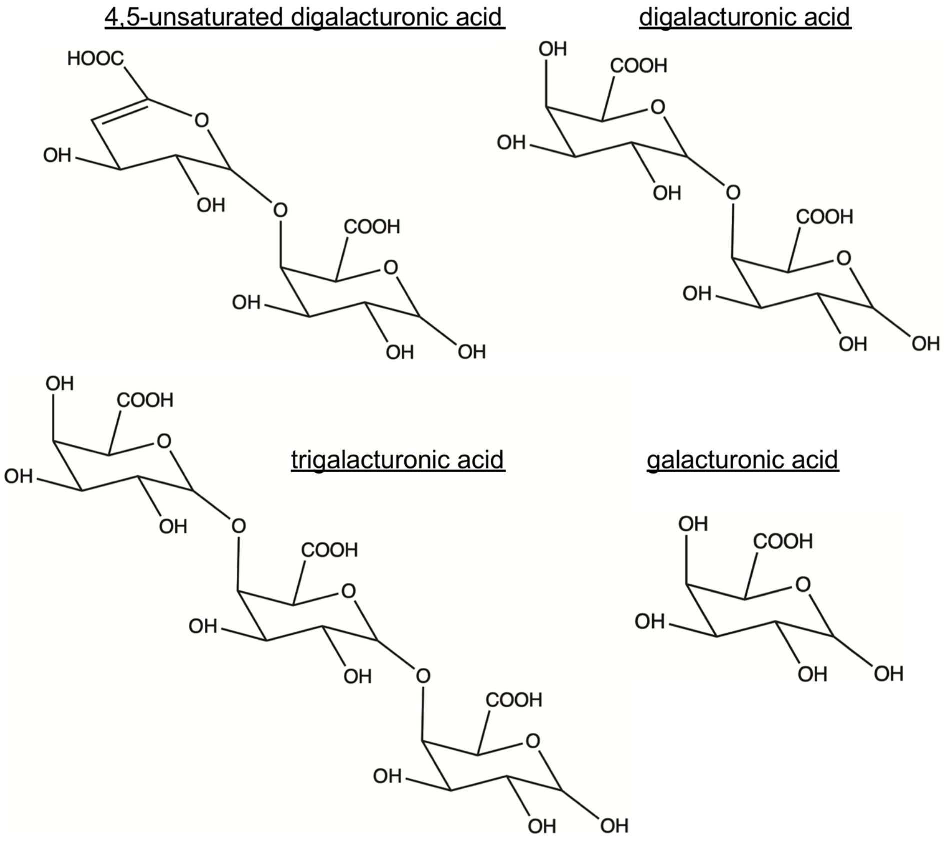

Figure S5. Structural diagrams of carbohydrates utilized in this study (generated using ChemDraw 20.1). Galacturonic acid is a monomer, whereas unsatdigalUA, digalUA, and trigalUA are oligos of galacturonic acid joined via α-1,4- glycosidic bonds.

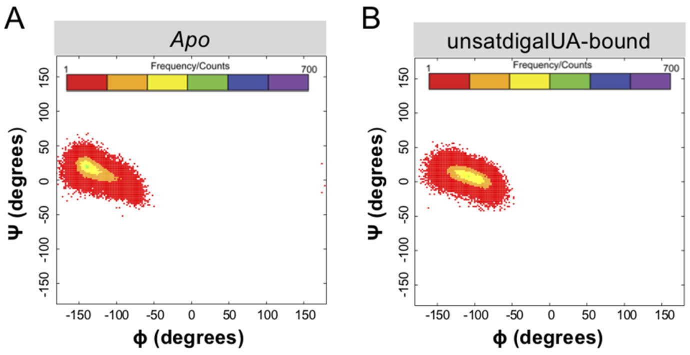

Figure S6. Amino acid Position 99 in TogB ($F_{Score2.0}$ = 0.40 ± 0.08 for unsatdigalUA) exhibits a moderate shift in dihedral angles upon ligand binding. Dihedral angles from representative apo (A) and unsatdigalUA-bound (B) 100 ns molecular dynamics simulations are shown. There is a clear population shift upon ligand binding (resulting in a mid-range $F_{Score2.0}$ value), however the residence times in the respective microstates is not reflected and occupancy may not be long enough to result in an environment change of a conjugated fluorophore that can contribute to the in vitro (ensemble) measurement.

Table S1. Comparison of kinetic properties between TogB D363C-MDCC, MalX A134C-MDCC[18], and HC PhoS A197C-MDCC[8].

	HC PhoS A197C-MDCC	MalX A134C-MDCC	TogB D363C-MDCC
Species detected	Phosphate	Maltooligosaccharides	Oligogalacturonides
ΔI upon ligand addition	+70% [8]	-29%[a] [18]	-60% [b]
k_{on} ($\mu M^{-1}s^{-1}$)	33 ± 2 [8]	20 ± 2 [a] [18]	18.6 ± 0.7 [b]
K_D (μM)	~0.100 [20]	0.190 ± 0.050 [a] [18]	1.3 ± 0.5 [b]

[a] *For Maltotriose detection*
[b] *For unsatdigalUA detection*
